# Lack of Atorvastatin Effect on Monocyte Gene Expression and Inflammatory Markers in HIV-1-infected ART-suppressed Individuals at Risk of non-AIDS Comorbidities

**DOI:** 10.20411/pai.v6i2.461

**Published:** 2021-08-13

**Authors:** Anjana Yadav, Andrew V. Kossenkov, Louise C. Showe, Sarah J. Ratcliffe, Grace H. Choi, Luis J. Montaner, Pablo Tebas, Pamela A. Shaw, Ronald G. Collman

**Affiliations:** 1 Department of Medicine; University of Pennsylvania Perelman School of Medicine, Philadelphia, PA; 2 Department of and Biostatistics and Epidemiology; University of Pennsylvania Perelman School of Medicine, Philadelphia, PA; 3 The Wistar Institute; Philadelphia, PA

**Keywords:** HIV/AIDS, Antiretroviral therapy, Atorvastatin, Monocyte, Gene expression, Cytokine, Inflammation, Immune activation

## Abstract

**Background:**

Many people living with HIV have persistent monocyte activation despite viral suppression by antiretroviral therapy (ART), which contributes to non-AIDS complications including neurocognitive and other disorders. Statins have immunomodulatory properties that might be beneficial by reducing monocyte activation.

**Methods:**

We previously characterized monocyte gene expression and inflammatory markers in 11 HIV-positive individuals on long-term ART (HIV/ART) at risk for non-AIDS complications because of low nadir CD4+ counts (median 129 cells/uL) and elevated hsCRP. Here, these individuals participated in a double-blind, randomized, placebo-controlled crossover study of 12 weeks of atorvastatin treatment. Monocyte surface markers were assessed by flow cytometry, plasma mediators by ELISA and Luminex, and monocyte gene expression by microarray analysis.

**Results:**

Among primary outcome measures, 12 weeks of atorvastatin treatment led to an unexpected increase in CCR2+ monocytes (*P*=0.04), but did not affect CD16+ or CD163+ monocytes, nor levels in plasma of CCL2/MCP-1 or sCD14. Among secondary outcomes, atorvastatin treatment was associated with decreased plasma hsCRP (*P*=0.035) and IL-2R (*P*=0.012). Treatment was also associated with increased total CD14+ monocytes (*P*=0.015), and increased plasma CXCL9 (*P*=0.003) and IL-12 (*P*<0.001). Comparable results were seen in a subgroup that had inflammatory marker elevations at baseline. Atorvastatin treatment did not significantly alter monocyte gene expression or normalize aberrant baseline transcriptional patterns.

**Conclusions:**

In this study of aviremic HIV+ individuals at high risk of non-AIDS events, 12 weeks of atorvastatin did not normalize monocyte gene expression patterns nor lead to significant changes in monocyte surface markers or plasma mediators linked to non-AIDS comorbidities.

**Clinical trial registration number:** NCT01600170

## INTRODUCTION

Despite effective viral suppression with antiretroviral therapy (ART), people with HIV infection (HIV/ART) experience an excess of serious non-AIDS comorbidities including HIV-associated neurocognitive disease (HAND), cardiovascular disease, and other affected organ systems. These serious non-AIDS events (SNAEs) are now a major cause of morbidity and mortality in ART-treated patients [1]. A common link among these SNAEs is that they are associated with persistent immune activation that is incompletely reversed in many patients even by prolonged complete viral suppression.

While persistent activation of T cells has received much attention, chronic myeloid cell activation appears to have a central role in the pathogenesis of these disorders [2, 3]. In untreated HIV infection, HAND results from the accumulation of macrophages in the CNS, derived from monocytes that traffic from blood, which release cytokines, small molecule excitotoxins, or viral proteins that lead to neurodegeneration, commonly resulting in HIV-associated dementia [4]. ART-suppressed individuals experience HAND that is less severe but frequent, with 20%-50% of treated patients having varying degrees of impairment [5, 6]. These individuals exhibit low-level neuroinflammation, with elevated CSF levels of sCD14 [7]. Several cytokines are elevated in the blood, including CCL2/MCP-1, which is chemotactic for monocytes and may play a role in monocyte tissue infiltration [8, 9], and sCD14, which is a marker of myeloid cell activation [10]. They also have an excess of blood monocytes that express CD16 (FcgammaRIII), CD163, and CCR2, which is the receptor for CCL2 [11–14]. Similar patterns have been reported for cardiovascular and other SNAEs in HIV/ART patients [15, 16].

The cause of persistent immune activation in HIV/ART patients is likely multifactorial, including microbial translocation from damaged gut mucosal barrier, residual HIV gene expression, co-infections such as cytomegalovirus, and disrupted immune cell homeostasis [17]. Chronic inflammation and SNAEs are especially prevalent and severe in people who begin ART with low CD4 levels [6, 18, 19]. Thus, this is a key group in which better understanding of mechanisms responsible for SNAEs is needed, as well as adjunctive therapies to downregulate drivers of these comorbidities. Statins, widely used in people living with HIV for lipid disorders, have pleiotropic immunomodulatory effects beyond those related to cholesterol lowering, which are mediated by lipid modifications of small signaling molecules [20]. Statins have been the focus of considerable interest as adjunctive therapy in HIV/ART patients [21–25]. *In vitro*, we [24] and others [26, 27] have shown that statins modulate monocyte activation by triggers that contribute to residual immune activation in HIV/ART patients.

To better understand the features of persistent monocyte activation in people at high risk for SNAEs, we recently reported an analysis of monocyte gene expression, monocyte surface molecules, and soluble plasma mediators in 11 long-term ART-suppressed individuals who had begun therapy with advanced disease (median nadir CD4 T-cell count 129 cells/uL) and had elevated hsCRP in the absence of end-organ comorbidities [28]. Compared with matched HIV-negative controls, HIV/ART patients had elevations in several plasma cytokines, the most prominent of which was CCL2/MCP-1 (>2-fold mean elevation; *P*=0.0001). Cluster analysis of plasma mediators showed that 6/11 HIV/ART participants clustered with controls, while 5 formed a distinct group, driven by IL-10, CCL11/Eotaxin, CXCL10/IP10, CCL2/MCP-1, CXCL9/MIG, and sIL2R. Monocyte subsets and surface markers did not differ significantly from controls, but transcriptomic analysis revealed dysregulation in monocytes of multiple genes, gene sets and pathway-linked to immune functions, including inflammation, immune cell development, and cell signaling.

In this study, we report the effect of treatment of these 11 HIV/ART individuals with atorvastatin or placebo for 12 weeks in a randomized, double-blind crossover study. We examined the effect of atorvastatin on soluble mediators, cell surface markers, and monocyte gene expression patterns. We also queried the effects in the subgroup of outlier individuals with coordinated elevations of inflammatory markers at baseline. We found a decrease in plasma hsCRP that did not meet *a priori* threshold for significance, and otherwise no compelling evidence for impact on monocyte surface or plasma markers, nor was there normalization of monocyte gene expression.

## MATERIALS & METHODS

### Study Design:

This study was a single-site randomized, double-blind, placebo-controlled crossover trial. The initial target enrollment was 30 participants, with an actual enrollment of 11. As previously described [28], enrollment criteria were ≥18 years of age with a plasma RNA <200 copies/mL for at least 6 months and receiving unchanged ART regimen ≥4 weeks prior to study entry, CD4+ T-cell count >100 cells/uL, and hsCRP greater than the upper limit of normal (>2mg/L). Initial criteria also stipulated a nadir CD4 count ≤250 cells/uL, which was adjusted to ≤350 partway through the study in an effort to expand enrollment. Exclusion criteria included hyperlipidemia or other clinical indications for statin use; history of atherosclerotic or other cardiac disease; diabetes; inflammatory or autoimmune conditions; hepatitis C infection or significant liver disease; use of corticosteroids, immunosuppressive therapy, or nonsteroidal anti-inflammatory drugs on a daily basis (Supplemental Table 1).

**Figure 1.**
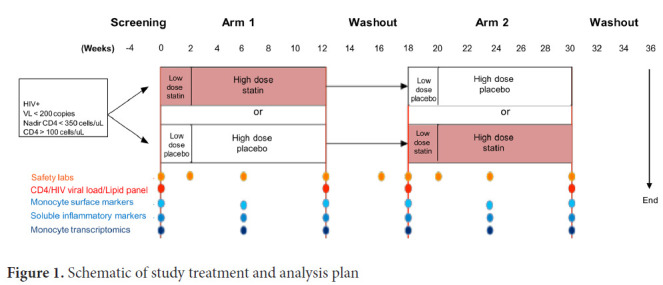
Schematic of study treatment and analysis plan

Following written informed consent, participants were randomized in arm 1 of the study to either atorvastatin at low dose for 2 weeks followed by high dose for an additional 10 weeks, or placebo, followed by a washout phase of 6 weeks (Figure 1). They were then switched to the opposite treatment in arm 2 of the study beginning at 18 weeks, with an identical schedule. For patients receiving protease inhibitors, the low and high dose atorvastatin dosages were 10 mg and 20 mg, respectively; for those on non-nucleoside reverse transcriptase inhibitors dosages were 40 mg and 80 mg; for all others, dosages were 20 mg and 40 mg. This study was approved by the University of Pennsylvania IRB (protocol #815512) and registered at clincaltrials.gov (NCT01600170).

### Flow cytometry:

Fresh whole blood was collected in EDTA tubes and stained with a cocktail of antibodies that included CD14-Pacific Blue, CD16-Apc-Cy7, CD163-PerCpCy5-5, CD3-BV570, CD4-PeCy5, CD8-PeTexRed, CX3CR1-Fitc, CCR2-Pe, CD38-PeCy7, HLA-DR-BV605 (clone L243), CD142-Pe, and PD-L1-APC. The antibody cocktail was added to 100uL of whole blood, vortexed gently, and incubated in the dark at room temperature for 20 minutes. RBC lysing buffer was then added, vortexed, and incubated for 10 minutes in the dark at room temperature. This was followed by centrifugation at 500*g* for 5 minutes to remove the supernatant. Cells were washed by adding 2 mL of FACS buffer and centrifuging at 500*g* for 5 minutes. Stained cells were suspended in 2% paraformaldehyde and stored at 4°C until acquisition on a modified LSRII (BD Immunocytometry Systems) and analyzed using FlowJo software (TreeStar). FACS analysis was done at weeks 0, 6, 12, and 18 (washout). The monocyte gating strategy is shown in Supplemental Figure 1.

### Plasma assays:

Levels of sCD14, CCL2, and sCD163 in EDTA-anticoagulated plasma were measured using Quantikine ELISA kits (R&D). Plasma LBP (LPS binding protein) was measured using Human LBP ELISA kit (Cell Sciences). All other cytokines and chemokines were measured using the Luminex multiplex human cytokine assay kit (cat no: LHC0009, Invitrogen). Plasma concentrations were measured at weeks 0, 2, 6, 12, and 18.

### Monocyte isolation:

Monocytes were purified for gene expression analysis at weeks 0, 6, 12, and 18 (washout). Blood was collected in EDTA tubes and peripheral blood mononuclear cells (PBMC) separated by Ficoll-gradient centrifugation. CD14+ monocytes were then isolated by negative selection with antibody-conjugated magnetic beads according to the manufacturer's instructions (Miltenyi Biotech), suspended in RNAlater (Ambion), and frozen at −80°C until RNA extraction. FACS analysis was done on each isolation and based on CD14+ staining, mean monocyte purity was 93% (range: 92%-94%).

### RNA isolation and Microarray Assays:

RNA was isolated using the Qiagen DNA/RNA mini kit (catalog no. 80204). RNA quality was confirmed using the Eukaryote Total RNA Nano Bioanalyzer (Agilent) assay, and all RNA used for analysis had a RIN (RNA Integrity number) >7. Total RNA (100 ng) was amplified with TargetAmp Nano-g Biotin-aRNA Labeling Kit (Epicentre; catalog no. TAN07924) to generate biotinylated labeled amplified RNA (aRNA). Biotin-labeled aRNA (750 ng) was hybridized to Illumina HumanHT-12V4 expression Beadchip (Illumina HumanHT-12 v4 Expression BeadChip Kit; catalog no. BD-103-0204). Illumina GenomeStudio software was used to export expression levels and detection of *P* values for each probe of each sample. Signal intensity data was quantile normalized and genes that showed an insignificant detection *P* value (*P*>0.05) in all samples were removed from further analysis, resulting in a set of 29,208 probes.

Expression level comparisons between 2 groups were done using 2-sample SAM algorithm [29]. False Discovery Rate (FDR) threshold was not used as cut-off criteria and only nominal *P* value thresholds (*P*<0.05, <0.01, and <0.001) were used to generate final gene sets. Gene set enrichment analysis for biological functions and canonical pathways was done using Ingenuity Pathway Analysis (IPA) software (QIAGEN). Additional enrichment analysis was done using GSEA [30] on pre-ranked by SAM genes and 1000 permutations to find significantly associated pathways (MSigDB set C2).

## STATISTICAL ANALYSES

Primary outcomes designated at time of study design were changes in percentages of CD14+ monocytes expressing CD16, CD163, or CCR2, and plasma CCL2/MCP-1 and sCD14. Secondary outcomes were defined as monocyte tissue factor (TF) and CX3CR1 expression, and plasma hsCRP, d-Dimer, and sCD163. All other measures as well as monocyte gene expression analysis were considered exploratory. The primary analysis for each outcome focused on a comparison of the change in response over the 12 weeks for each treatment under a 2x2 crossover design. Mixed effects models were used to estimate (a) within-participant treatment differences, (b) trends over time, and (c) differences at each week (0, 2, 6, 12) on treatment. Log-transformations were applied as necessary to meet normality assumption. All models were adjusted for potential period and sequence effects. The treatment effects for primary outcomes were pre-specified to use a *P*<0.01, whereas secondary and exploratory outcomes used a *P*<0.05 for statistical significance. *P* values are unadjusted for multiple comparisons. Statistical significance for lipid level comparisons at 12 weeks was calculated using Wilcoxon matched-pairs signed rank 2-tailed paired *t*-test.

## RESULTS

### Participant characteristics and treatment safety:

HIV+ participants were virally suppressed on long-term ART, without end-organ comorbidities or clinical indications for statin therapy, but at high risk of SNAEs based on low nadir CD4 counts and persistent inflammation based on elevated hsCRP. Eleven participants were enrolled (Table 1), with a median CD4 count at enrollment of 529 cells/uL (range 342-1062) and nadir of 129 cells/uL (range 24-233). Participants were receiving ART for a mean of 7.4 years (range 3-13 years) and were virally suppressed with all HIV-1 viral load levels <200 for 6 months prior to entry, and most below the lower level of quantitation (<20 copies/mL) at enrollment. Participants included 8 men and 3 women; 10 were Black and 1 White; and 2 were smokers. The study design is shown in Figure 1.

**Table 1:** Demographic and clinical information on participants

Participants	N=11
Age (median; range)	49 (27-60)
Gender:	
Male (n; %)	8 (72%)
Female (n; %)	3 (23%)
Race:	
Black (n; %)	10 (91%)
White (n; %)	1 (9%)
Smoker (n; %)	2 (18%)
CD4 count at entry (median; range) (cells/uL)	529 (172-1062)
CD4 count nadir (median; range) (cells/uL)	129 (24-233)
Duration of ART (median; range) (years)	7(3-13)
Viral load at entry (median; range) (copies/mL)	<20 (<20-137)
hsCRP (median; range) (mg/L)	4 (1.6*-9.7)
Total Cholesterol (median; range) (mg/dL)	170 (106-239)
LDL Cholesterol (median; range) (mg/dL)	103 (45-152)

* One participant fulfilled enrollment criteria of hsCRP ≥ 2mg/dL at screening visit but was below that level at entry

As shown in Supplemental Figure 2, 12 weeks of atorvastatin treatment resulted in a significant reduction in LDL (*P*=0.0117) as well as total cholesterol levels (*P*=0.0195). There were no adverse events (AE) that were deemed to be treatment-related. HIV-1 plasma viral load and CD4+ T-cell counts were measured at entry and week 12. Three participants had detectable viral loads of 137, 114, and 106 copies/mL at entry that subsequently became undetectable (<20 copies/mL) at 12 weeks, and there were no changes in percentage of CD4+ T cells associated with treatment (data not shown).

### Effect of atorvastatin on monocyte cellular markers associated with immune activation:

Whole blood was stained for monocyte cellular markers, and evaluated for changes in expression at 12 weeks over baseline within each treatment group (placebo and atorvastatin; Table 2; columns 1 and 2), and differences over 12 weeks between placebo and atorvastatin groups (Table 2; column 3 and 4). Primary outcome measures were monocyte expression of CD16, CD163, and CCR2. CD16 and CD163 are cell populations that have been linked to SNAEs, particularly neuro-cognitive impairment, while CCR2 is the receptor for CCL2/MCP-1, which is elevated in chronic HIV/ART participants and thought to be a driver of myeloid cell accumulation in the brain and other tissues.

**Table 2.** Change in monocyte subsets over 12 weeks of atorvastatin or placebo treatment

Monocyte subset	Placebo (fold change over 12 weeks)	Atorvastatin (fold change over 12 weeks)	Difference in fold change between groups	Raw *P* value (between 2 groups)
CD14+CD16+	1.51	1.14	0.75	0.385
CD14+CD163+	−5.14	−0.06	5.08	0.350
CD14+CCR2+	0.78	1.60	2.05	**0.040**
CD14+CD16+CD163+	1.15	1.03	0.90	0.801
CD14+TF+	1.52	1.18	0.78	0.625
CD14+CX3CR1 +	1.02	0.92	0.90	0.816
CD14+CD38+	1.04	0.98	0.94	0.619
CD14+PDL1 +	0.60	0.59	0.99	0.971
Total CD14+	0.93	1.25	1.35	**0.015**

Data were log transformed for normal distribution and are expressed as fold change, except CD14+CD163+, which are shown as mean difference of the percentage of CD163+ monocytes.

**Figure 2.**
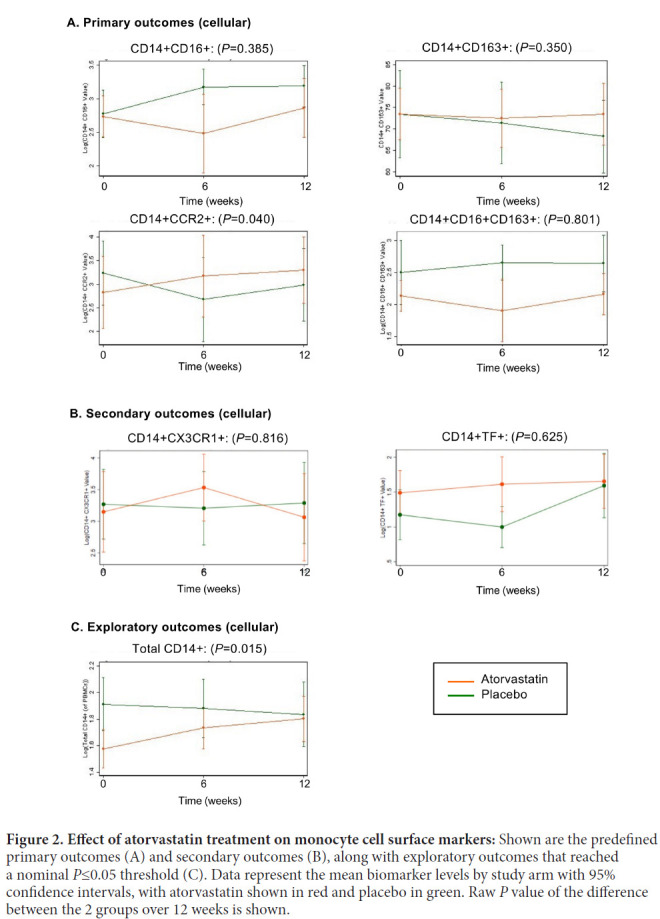
**Effect of atorvastatin treatment on monocyte cell surface markers:** Shown are the predefined primary outcomes (A) and secondary outcomes (B), along with exploratory outcomes that reached a nominal *P*≤0.05 threshold (C). Data represent the mean biomarker levels by study arm with 95% confidence intervals, with atorvastatin shown in red and placebo in green. Raw *P* value of the difference between the 2 groups over 12 weeks is shown.

We found an increase in the proportion of monocytes expressing CCR2 (CD14+CCR2+) following 12 weeks of atorvastatin compared to placebo (2-fold difference; *P*=0.04), although this did not reach the pre-specified *P*≤0.01 threshold for significance (Figure 2, Table 2). This increase was counter to anticipation that atorvastatin would decrease CCR2 expression. There were no significant differences between statin and placebo treatment in CD16+, CD163+, or CD16+CD163+ monocyte populations. Similarly, there were no significant differences between atorvastatin and placebo in secondary outcome measures (CD14+CX3CR1+ and CD14+TF+ monocytes) (Figure 2, Table 2). Among exploratory outcomes, 12 weeks of statin treatment was associated with an increase in total CD14+ monocytes compared to placebo (1.35-fold change; *P*=0.015).

### Effect of atorvastatin on plasma markers associated with monocyte activation:

Soluble mediators were measured in plasma by ELISA and Luminex assay. Primary outcomes were levels of CCL2/MCP-1, due to the potential role for CCL2/MCP-1 in monocyte tissue migration, and sCD14, reflecting monocyte activation by bacterial products. CCL2/MCP-1 was also the most elevated marker in baseline in these HIV/ART patients compared to controls [28]. Secondary outcomes were hsCRP, sCD163, and D-dimer.

**Figure 3.**
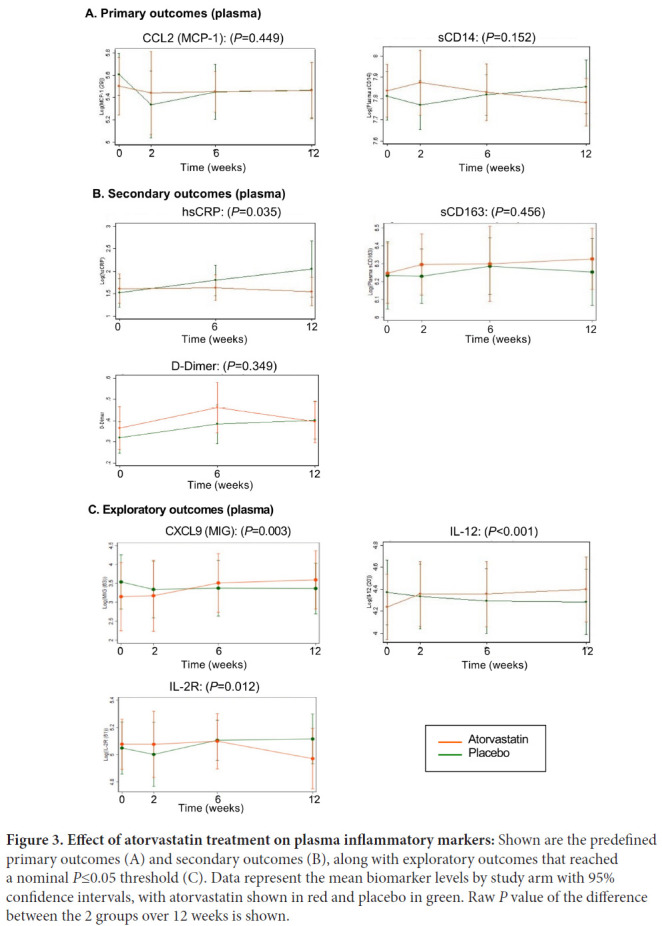
**Effect of atorvastatin treatment on plasma inflammatory markers:** Shown are the predefined primary outcomes (A) and secondary outcomes (B), along with exploratory outcomes that reached a nominal *P*≤0.05 threshold (C). Data represent the mean biomarker levels by study arm with 95% confidence intervals, with atorvastatin shown in red and placebo in green. Raw *P* value of the difference between the 2 groups over 12 weeks is shown.

There were no significant differences in CCL2/MCP-1 or sCD14 levels after 12 weeks of atorvastatin compared with placebo treatment (Figure 3 and Table 3). Among secondary outcomes, there was a significant difference between statin-treated and placebo-treated patients in hsCRP, although this resulted mainly from an increase over 12 weeks in placebo but not atorvastatin recipients (fold-difference 0.55; *P*=0.035). No significant differences were seen for sCD163 or D-dimer.

**Table 3.** Change in plasma markers over 12 weeks of atorvastatin or placebo treatment

Soluble markers	Placebo (fold change over 12 weeks)	Atorvastatin (fold change over 12 weeks)	Difference in fold change between groups	Raw *P* value (between 2 groups)
CCL2 (MCP-1)	0.87	0.96	1.11	0.449
sCD14	1.04	0.95	0.91	0.152
hsCRP	1.70	0.94	0.55	0.035
sCD163	1.02	1.08	1.06	0.456
D-dimer	0.09	0.95	0.86	0.349
CCL3 (MIP-1a)	0.78	0.67	0.86	0.559
CCL4 (MIP-1b)	0.88	1.04	1.18	0.381
CCL5 (RANTES)	1.05	0.94	0.90	0.196
CXCL9 (MIG)	0.84	1.56	1.85	0.003
CXCL10 (IP-10)	0.84	0.99	1.17	0.554
CCL11 (Eotaxin)	0.84	1.00	1.19	0.109
IFN-a	0.98	0.89	0.92	0.240
IFN-g	0.99	0.96	0.96	0.306
IL-2	0.99	0.88	0.89	0.516
IL-6	1.25	1.04	0.83	0.410
IL-8	0.59	0.95	1.62	0.133
IL-10	0.95	1.14	1.20	0.290
IL-12	0.92	1.18	1.28	<.001
IL-1Ra	1.39	1.57	1.13	0.769
IL-2R	1.07	0.90	0.84	0.012
LBP	1.28	1.07	0.84	0.173

Data were log transformed for normal distribution and are expressed as fold change, except D-dimer, which is shown as mean difference of levels in plasma.

Among additional soluble markers that were measured in exploratory analysis, we found differences in statin versus placebo treatment in CXCL9 (MIG) (1.85-fold difference *P*=0.003), IL-12 (1.28-fold difference; *P*<0.001), and IL-2R (0.84-fold difference; *P*=0.012) (Figure 3, Table 3). Notably, the directionality of differences for CXCL9 and IL-12 reflected higher levels following atorvastatin compared with placebo treatment. Furthermore, among the exploratory mediators tested that did not show significant differences between treatments, nominal differences trending towards higher levels following statin compared to placebo were seen for most (Table 3).

### Atorvastatin effect in HIV/ART individuals with elevated inflammatory biomarkers at baseline.

Our previous analysis of these HIV/ART individuals prior to atorvastatin treatment (baseline week 0) compared with matched HIV-negative controls found that HIV/ART individuals as a group had significantly elevated plasma CCL2/MCP-1, CXCL9/MIG and sIL2R, which were correlated with each other [28]. Principal component analysis of the full set of cytokines measured revealed that 6 of the 11 HIV/ART participants clustered with controls, while 5 formed a distinct group, driven by elevations in CCL2/MCP-1, CXCL9/MIG, and sIL2R as well as IL-10, CCL11/Eotaxin, and CXCL10/IP-10. This subgroup was older than those who clustered with controls (mean ± SE: 57.6±1.6 years versus 36.4±2.4 years respectively; *P*=0.012, Mann-Whitney test), but did not differ in current or nadir CD4 count or other features. Since that analysis suggested a pattern of coordinated immune activation in a subset of HIV/ART participants, we sought to investigate the effects of atorvastatin treatment within this sub-group, though recognizing the limitation of the small number individuals in the group.

As shown in Table 4, there were no significant differences between atorvastatin and placebo treatment in primary or secondary outcome measures of monocyte surface marker expression within this subgroup with coordinated inflammatory marker elevations at baseline. Like the entire HIV/ART group, this subgroup also had a significant increase in total CD14+ monocytes with 12 weeks of atorvastatin compared with placebo treatment (*P*<0.001). There was also a significant increase in PDL1+ monocytes in the subgroup (*P*=0.019), which was not observed in the entire HIV+/ART+ group.

**Table 4.** Effect of atorvastatin in a subset of participants identified as plasma marker outliers at baseline

Monocyte subset	Placebo (n=5) (fold change over 12 weeks)	Atorvastatin (n=5) (fold change over 12 weeks)	Difference in fold change between groups	Raw *P* value (between 2 groups)
CD14+CD16+	0.95	1.21	1.28	0.607
CD14+CD163+	1.86	−6.00	−7.86	0.255
CD14+CCR2+	0.97	1.64	1.69	0.188
CD14+C-D16+CD163+	0.52	1.13	2.2	0.299
CD14+TF+	0.83	1.34	1.61	0.624
CD14+CX3CR1 +	1.77	1.02	0.58	0.364
CD14+CD38+	1.08	0.92	0.85	0.410
CD14+PDL1 +	−16.11	−0.06	16.05	**0.019**
Total CD14+	0.79	1.35	1.71	**<0.001**
**Soluble markers**				
CCL2 (MCP-1)	0.83	1.01	1.22	0.307
sCD14	1.04	0.87	0.84	**0.016**
hsCRP	1.39	0.83	0.60	0.238
sCD163	1.02	1.05	1.03	0.816
D-dimer	0.10	0.04	−0.06	0.317
CCL3 (MIP-1a)	0.83	0.96	1.15	0.254
CCL4 (MIP-1b)	0.79	1.2	1.2	0.108
CCL5 (RANTES)	1.07	0.88	0.82	0.220
CXCL9 (MIG)	0.82	1.54	1.88	**0.002**
CXCL10 (IP-10)	0.71	1.22	1.73	0.192
CCL11 (Eotaxin)	0.90	1.08	1.19	0.190
IFN-g	0.95	0.98	1.03	0.625
IFN-a	0.96	0.88	0.91	0.126
IL-2	0.96	0.95	0.99	0.973
IL-6	1.02	0.79	0.77	0.301
IL-8	0.35	1.02	2.95	**0.067**
IL-10	0.90	1.26	1.41	0.353
IL-12	0.94	1.23	1.3	**0.018**
IL-1Ra	1.39	3.44	2.48	0.193
IL-2R	1.06	0.91	0.86	0.280
LBP	1.49	1.05	0.71	**0.063**

Data were log transformed for normal distribution and are expressed as fold change, except CD14+CD163+, CD14+PDL1+ and D-dimer, which are shown as mean difference.

Analysis of plasma markers in this subgroup (Table 4) revealed a decrease in sCD14 with atorvastatin compared to placebo following 12 weeks of treatment (0.84-fold change; *P*=0.016). Neither CCL2/MCP-1 nor any secondary outcome measures reached statistical significance. Within the expanded group of exploratory measures, significant changes were seen for IL-12 (*P*=0.018), and CXCL9/MIG (*P*=0.002), both of which were in the direction of higher levels following atorvastatin treatment compared with placebo. As in the larger group, most exploratory markers that did not reach statistical significance showed nominal differences that trended towards higher levels following atorvastatin compared to placebo treatment.

### Relationship between primary outcomes and baseline characteristics or lipid changes with atorvastatin:

There was no significant relationship between change in plasma CCL2 or sCD14, or monocyte CD16, CD163, or CCR2, and baseline hsCRP, CD4+ T-cell counts, or total or LDL cholesterol (data not shown). We also asked if primary outcomes correlated with evidence of atorvastatin's effect on lipids or hsCRP, but found no significant relationship between primary outcome measures and change in total or LDL cholesterol or hsCRP (data not shown).

### Pathway analysis of atorvastatin treatment effect on monocyte gene expression:

We investigated monocyte gene expression changes using Ingenuity Pathway Analysis (IPA), to identify potential pathways impacted by atorvastatin. Using IPA, we applied pairwise comparisons to identify gene expression changes between atorvastatin and placebo group at weeks 0 (baseline), 6, 12, and 18 (washout). We also queried changes at 6 weeks and 12 weeks within the atorvastatin group compared to week 0.

**Figure 4.**
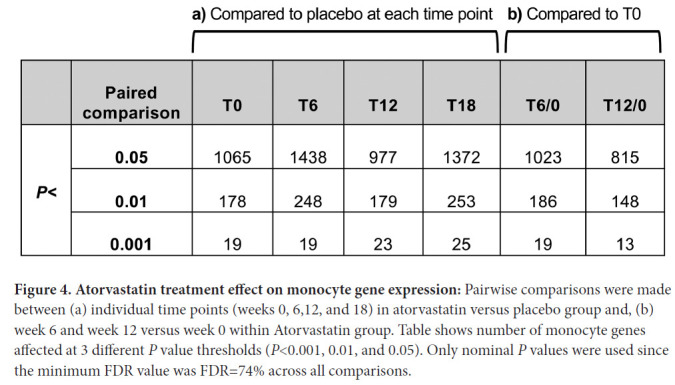
**Atorvastatin treatment effect on monocyte gene expression:** Pairwise comparisons were made between (a) individual time points (weeks 0, 6,12, and 18) in atorvastatin versus placebo group and, (b) week 6 and week 12 versus week 0 within Atorvastatin group. Table shows number of monocyte genes affected at 3 different *P* value thresholds (*P*<0.001, 0.01, and 0.05). Only nominal *P* values were used since the minimum FDR value was FDR=74% across all comparisons.

The minimum FDR across all comparisons was only 74% (data not shown). We therefore queried these changes using different nominal *P* value thresholds (<0.05, <0.01, and <0.001). As shown in Figure 4 (left side of table), after 6 weeks of treatment with atorvastatin as compared to placebo there were 1438, 248, and 19 monocyte genes affected at these nominal *P* value thresholds, respectively. At 12 weeks of treatment, there were 977, 179, and 23 affected genes at *P*<0.05, *P*<0.01 and *P*<0.001. The numbers of genes differing at 6 and 12 weeks between atorvastatin and placebo treatment at these thresholds were similar to the numbers of genes that differed between groups before treatment (week 0) and after the 6-week washout period (week 18). This observation raises the possibility that differences might be stochastic and unrelated to treatment, which is consistent with the lack of differences between treatment groups that pass correction for multiple testing.

We also analyzed change in gene expression within the atorvastatin group by comparing week 6 and week 12 to week 0 at the same *P* value thresholds, as shown in Figure 4 (right side of table). In this comparison, after 6 weeks of treatment there were 1023 genes whose expression was changed compared to baseline, which decreased to 815 genes after 12 weeks of treatment at *P*<0.05. A similar trend was seen at *P*<0.01 and *P*<0.001. Both between-group and within-group comparisons showed more monocyte genes with altered expression levels after 6 weeks of atorvastatin treatment than after 12 weeks of treatment. Finally, when the same analysis was carried out using an unpaired version of the comparisons, fewer significant genes were identified than with the paired analysis (data not shown), suggesting a large participant effect.

Ingenuity Pathway Analysis of the differentially expressed probes (*P*<0.01) identified affected functions and pathways at weeks 6 and 12 in the atorvastatin group (Table 5). Of the 500 functions queried, only 5 changed state significantly 6 weeks after statin treatment; of the 310 canonical pathways queried, only 1 was significantly altered. Similarly, after 12 weeks after statin treatment only 2 functions changed state and no pathways were affected (Table 5).

**Table 5.** Ingenuity Pathway Analysis functions altered at week 6 and week 12 of atorvastatin treatment

Week 6 versus Week 0			
Function	*P* value	Z	State
HIV infection	0.00000198	−2.7	Decreased
infection by HIV-1	0.000031	−2.7	Decreased
infection by RNA virus	0.0000374	−2.1	Decreased
infection of cells	0.000159	−2.2	Decreased
organismal death	0.00165	**4.17**	**Increased**

Pathway	*P* value	Z	State
mTOR Signaling	0.000776247	−2.5	Inhibited

Week 12 versus Week 0			
Function	*P* value	Z	State
hypoplasia of heart ventricle	0.00446	**2**	**Increased**
cell viability of tumor cell lines	0.013	−2.2	Decreased

IPA functions altered at weeks 6 and 12 compared to week 0 within the atorvastatin treatment arm, based on genes as shown in Figure 4b using cutoff criteria *P*<0.01.

We also applied Gene Set Enrichment Analysis (GSEA), which employs the whole gene expression dataset ranked in order of difference between the atorvastatin and placebo groups to identify gene expression effects of atorvastatin treatment (Supplemental Table 2 and Supplemental Table 3). Drug effects were evaluated at week 6 and week 12 compared to baseline (week 0) within the treatment group. After 6 weeks of atorvastatin, 399 gene sets were enriched at nominal *P*<0.01 and 1434 gene sets at nominal *P*<0.05. Only one gene set was enriched at FDR<25% (Supplemental Table 2). Week 12 analysis showed enrichment of 443 gene sets at nominal *P*<0.01 and 1461 gene sets at nominal *P*<0.05. No gene sets were significant at FDR<25% at 12 weeks (Supplemental Table 3). Most of the enriched gene sets (pathways and functions) were immune or metabolism related.

There was no overlap of gene sets at weeks 6 and 12 either by IPA or by GSEA, suggesting that the atorvastatin treatment effect on gene expression was not large enough to consistently modify any gene sets at both timepoints.

### Effect of atorvastatin on a priori genes of interest:

We then tested the atorvastatin effects at week 6 and 12 compared to week 0 on a set of monocyte immune activation genes of interest, which included monocyte surface molecules and secreted factors [28]. As shown in Table 6, none of the genes showed a significant fold-change either at week 6 or week 12 of atorvastatin compared to week 0. Furthermore, at the nominal level, changes were modest and often differed in direction at weeks 6 and 12.

**Table 6.** Fold change in monocyte genes of *a priori* interest following 6 and 12 weeks of atorvastatin treatment

Gene Symbol	T6 / 0 (Fold)	T6 / 0 (*P* value)	T12 / 0 (Fold)	T12 / 0 (*P* value)
FCGR3A (CD16)	1.04	0.42	−1.01	0.76
FCGR3B (CD16)	1.19	0.42	−1.11	0.49
CD163	−1.13	0.1	−1.01	0.7
CCR2	−1.1	0.51	−1.09	0.52
F3 (TF)	−1.04	0.47	−1.05	0.15
CX3CR1	1.07	0.23	−1.07	0.14
CD14	1	0.99	−1.03	0.49
CCL2	1.01	0.6	1.28	0.44
CXCL9 (MIG)	−1.11	0.75	−1.09	0.81
CXCL10 (IP10)	−1.09	0.99	1.03	0.31
IL1B	1.14	0.92	1.06	0.67
IL8	1.24	0.57	−1.06	0.41
IL10	1.07	0.38	1.12	0.4

### Effect of atorvastatin treatment on monocyte functions and pathways that were altered at baseline in HIV/ART compared with HIV-negative participants:

In our previous cross-sectional study of these HIV/ART individuals at baseline, we carried out a discovery analysis of monocyte gene expression differences compared to HIV-negative control participants [28]. Here we asked whether the genes that differed at baseline in these HIV/ART participants compared to HIV-negative control participants might be impacted by atorvastatin treatment. Table 7 shows the functions identified by IPA in that study (using cutoff criteria FDR<10%), which were almost all downregulated in HIV/ART compared with control participants. Of the 64 functions that differed at baseline between HIV/ART versus HIV-negative participants, only 3 were significantly affected by atorvastatin treatment at week 6, and none at week 12. In addition, there were 11 pathways identified by IPA that differed at baseline, all of which were downregulated in the HIV/ART compared to control group, and none were affected by atorvastatin treatment (data not shown). Thus, these data do not support the idea that atorvastatin treatment results in normalization of monocyte gene expression patterns in HIV/ART patients.

**Table 7.** Effect of atorvastatin on gene pathways that were significantly altered in HIV/ART compared to HIV-negative individuals at baseline

Functions	T0 State	T0 vs healthy (Z-value)	6 wk atorvastatin (*P* value)	12 wk atorvastatin (*P* value)
proliferation of cells	Decreased	−4.477		
quantity of blood cells	Decreased	−3.895		
quantity of leukocytes	Decreased	−3.856		
quantity of cells	Decreased	−3.751		
migration of cells	Decreased	−3.748		
quantity of mononuclear leukocytes	Decreased	−3.697		
cell movement	Decreased	−3.629		
quantity of lymphocytes	Decreased	−3.587	0.0098	
development of blood cells	Decreased	−3.43		
development of leukocytes	Decreased	−3.43		
development of lymphocytes	Decreased	−3.43		
homeostasis of leukocytes	Decreased	−3.309		
quantity of T lymphocytes	Decreased	−3.308		
flux of ion	Decreased	−3.265		
proliferation of T lymphocytes	Decreased	−3.254		
proliferation of lymphocytes	Decreased	−3.241	0.00324	
phosphorylation of protein	Decreased	−3.22		
proliferation of immune cells	Decreased	−3.187		
T cell development	Decreased	−3.167		
proliferation of blood cells	Decreased	−3.14		
flux of Ca2+	Decreased	−3.113		
quantity of metal ion	Decreased	−2.934		
production of reactive oxygen species	Decreased	−2.825		
leukocyte migration	Decreased	−2.804		
synthesis of reactive oxygen species	Decreased	−2.787		
quantity of CD4+ T-lymphocytes	Decreased	−2.664		
killing of cells	Decreased	−2.655		
degranulation of cells	Decreased	−2.647		
cell movement of leukocytes	Decreased	−2.58		
cell transformation	Decreased	−2.565		
formation of cytoskeleton	Decreased	−2.557		
differentiation of blood cells	Decreased	−2.538		
immune response of cells	Decreased	−2.522		
airway hyperresponsiveness	Decreased	−2.449		
interaction of T lymphocytes	Decreased	−2.434		
interaction of cells	Decreased	−2.434		
influx of Ca2+	Decreased	−2.412		
differentiation of leukocytes	Decreased	−2.406		
phosphorylation of L-amino acid	Decreased	−2.401		
maturation of T lymphocytes	Decreased	−2.4		
formation of filaments	Decreased	−2.384		
activation of cells	Decreased	−2.374		
cellular homeostasis	Decreased	−2.373	0.00115	
differentiation of mononuclear leukocytes	Decreased	−2.318		
proliferation of tumor cell lines	Decreased	−2.279		
binding of cells	Decreased	−2.257		
maturation of cells	Decreased	−2.252		
development of cytoplasm	Decreased	−2.242		
cytolysis of tumor cells	Decreased	−2.219		
differentiation of lymphocytes	Decreased	−2.214		
eosinophilia	Decreased	−2.213		
binding of T lymphocytes	Decreased	−2.2		
phosphorylation of L-tyrosine	Decreased	−2.191		
mobilization of cells	Decreased	−2.186		
polarization of T lymphocytes	Decreased	−2.183		
formation of actin stress fibers	Decreased	−2.155		
cell survival	Decreased	−2.125		
generation of lymphocytes	Decreased	−2.084		
mobilization of Ca2+	Decreased	−2.064		
cell viability	Decreased	−2.052		
proliferation of hematopoietic cell lines	Decreased	−2.037		
maturation of blood cells	Decreased	−2.03		
reorganization of cytoskeleton	Decreased	−2		
hypoplasia of thymus gland	**Increased**	**2.596**		
hypoplasia of thorax	**Increased**	**2.95**		

Monocyte functions identified by IPA as altered in HIV/ART compared to controls at baseline (cutoff criteria FDR<10%).

## DISCUSSION

In this study we tested the effect of atorvastatin on monocyte surface marker expression, plasma inflammatory markers, and monocyte gene expression in a group of HIV-infected virally suppressed individuals at high risk for serious non-AIDS comorbidities such as neurocognitive and cardiovascular disease, but without established comorbidities or clinical indications for statin use. We did not find decreases in monocyte activation markers or plasma cytokines that reached the pre-specified level for significance, nor evidence from secondary or exploratory markers to support a beneficial effect. Furthermore, there were no changes in monocyte gene expression that reached significance threshold, nor evidence suggesting normalization of aberrant monocyte gene expression profiles.

A novel aspect of our study was the prospective investigation of monocyte gene expression, testing the idea that atorvastatin effects on gene sets or pathways *in vivo* might give insight into effects beyond that evident in surface or soluble markers. However, the differences seen between atorvastatin and placebo groups, and between baseline and weeks 6 and 12 of atorvastatin, were modest and had low statistical power after correction for false discovery. Furthermore, there was no overlap between gene expression changes at week 6 and week 12. We also did not observe any meaningful effect of atorvastatin on an *a priori* set of immune activation-associated genes. Finally, when we focused specifically on gene functions and pathways that we previously found were dys-regulated in HIV/ART compared with control participants, there was no evidence that they were impacted as a group by atorvastatin. In contrast, a recent retrospective study of HIV+ ART-treated women with subclinical cardiovascular disease reported that treatment with statins for clinical indications was associated with downregulation of inflammatory genes in classical monocytes [25]. Thus, in HIV/ART participants without clinical indications for statin use, we find no evidence for monocyte gene expression effect of atorvastatin that exceeds random inter-participant and intra-participant variability, or that treatment shifts monocyte gene expression closer towards “normal” patterns.

Our soluble marker primary outcome measures were sCD14 and CCL2/MCP-1. An indicator of monocyte activation, sCD14, is elevated in HIV/ART patients, and has been widely studied as a marker of monocyte inflammation and correlate of SNAEs [31, 32]. CCL2/MCP-1 is also elevated in HIV/ART patients, and of interest because it is involved in myeloid transmigration into tissue and the pathogenesis of end-organ comorbidities [28, 33]. At baseline, CCL2/MCP-1 was the most significantly elevated plasma marker in these participants compared to HIV-negative controls (mean 298 versus 139 pg/mL; *P*=0.0001) [28]. However, neither sCD14 nor CCL2/MCP-1 were significantly reduced by atorvastatin. Among secondary endpoints, we did find a reduction with atorvastatin in hsCRP (*P*=0.035). On the other hand, we saw increases in multiple other cytokines in the exploratory panel, including CXCL9 (MIG) (*P*=0.003) and IL-12 (*P*<0.001), and trends for several others. Thus, there is no compelling evidence for beneficial effect of atorvastatin on soluble inflammatory markers in these participants.

Our cellular primary outcome markers were monocyte expression of CD16 and CD163, which are indicators of monocyte activation and linked to comorbidities including neurocognitive disease, as well as CCR2, which is the receptor for CCL2/MCP-1 and thus functionally important. Although not reaching the pre-specified significance threshold, atorvastatin treatment was associated with a change in CCR2+ monocytes compared with placebo (*P*=0.040), but this was due to increased expression and contrary to the direction of effect anticipated. Finally, we saw an increase in total monocytes (*P*=0.015), the significance of which is uncertain. Thus, these data do not support a beneficial effect of atorvastatin on monocyte subsets and surface expression profiles.

To ask whether atorvastatin might affect inflammatory markers in individuals at particularly high risk for SNAEs (beyond the selection criteria used here of nadir CD4 and hsCRP), we carried out a post-hoc subgroup analysis. In our previous investigation of these individuals at baseline compared to HIV-negative controls [28], integrated analysis of multiple plasma inflammatory markers revealed that about half of participants (6/11) clustered with controls, while the others (5/11) comprised an outlier group that was driven by elevations in CCL2/MCP-1, CXCL9/MIG and sIL2R, IL-10, CCL11/Eotaxin, and CXCL10/IP10. Analysis of this subgroup showed that atorvastatin was associated with decreased sCD14 (*P*=0.016) but also with increased CXCL9 (*P*=0.002) and IL-12 (*P*=0.018). The exact significance of the latter 2 observations is not known. As found in the whole cohort, several exploratory cytokines of interest were increased. Thus, evidence for benefit was lacking even in this very highly selected subgroup.

We selected atorvastatin because it is inexpensive, widely available, has only modest pharmaco-kinetic interactions with ART drugs, and would thus be particularly useful in resource-limited settings, where many HIV+ individuals begin ART at advanced disease [34] and would benefit from effective adjunctive therapy. Our results contrast with a large study of rosuvastatin, which reported significant decreases in monocyte and T-cell markers as well as plasma markers linked to increased SNAEs in HIV/ART individuals [21, 23]. In addition, a large study investigating effects of pitavastatin and pravastatin on markers of immune activation found significant reduction in sCD14 with pitavastatin but not pravastatin treatment, although monocyte cellular markers were not investigated [35]. On the other hand, 2 recently reported studies examined atorvastatin in HIV/ART patients. A multicenter study of atorvastatin treatment did not find significant effects on T-cell or monocyte activation markers, including sCD14, sCD16, CCL2/MCP-1, or in monocyte surface markers [36]. Another report examined the effect of atorvastatin in combination with raltegravir in people living with HIV and who were previously receiving protease inhibitor [37]. That study did not examine monocyte markers, but found no significant effect on T-cell activation nor plasma markers such as sCD14, CRP, D-dimer, and others. Our study is concordant with those results, and in addition, demonstrates lack of effect on monocyte gene expression patterns. Taken together, these data suggest that despite sharing broad class mechanisms, different statins may have fundamentally different effects on residual immune activation in HIV/ART individuals.

The immunomodulatory activity of statins results mainly from inhibiting the isoprenylation of small GTPase signaling molecules, thereby inactivating them, as well as potential direct effects on nuclear transcription factors [38, 39]. Statins vary in potency, pharmacokinetics, metabolism pathways and hydrophilic versus lipophilic properties, but differences in immunomodulatory activity are not well explored. Atorvastatin and pitavastatin are both lipophilic, whereas pitavastatin and rosuvastatin are both hydrophilic. Thus, the reasons that agents might differ in anti-inflammatory effects in ART-treated HIV+ people remain to be determined.

These *in vivo* findings stand in contrast to our previous *in vitro* studies demonstrating anti-inflammatory effects of atorvastatin and simvastatin on activation-associated monocyte surface markers and soluble factors [24]. We found that both atorvastatin and simvastatin reduced the proportion of CD16+ monocytes, as well as expression of CD163. Additionally, statin treatment reduced production of CCL2/MCP-1 and several other inflammatory factors upon LPS stimulation, as well as monocyte chemotaxis in response to CCL2/MCP-1. The contrast between promising *in vitro* effects and lack of *in vivo* effect highlights the complexity of factors driving myeloid activation in HIV/ART individuals. Clinical factors such as vitamin D status and smoking have been proposed to modify the effect of statin immunomodulation in HIV/ART patients [40, 41]. We do not have data on vitamin D levels, and only 2 of our participants were smokers. Whether these or other unrecognized confounders contributed to the lack of atorvastatin effect is uncertain.

A limitation of our study is the small sample size, which did not achieve its recruitment goal of 30 and is underpowered to reach the stringent statistical endpoints established in advance. Nevertheless, even with this caveat, non-significant trends did not generally favor reductions in cellular or soluble inflammatory markers with atorvastatin. It is possible that a larger cohort size might have allowed identification of atorvastatin effects on monocyte gene expression, although the lack of gene functions and pathways that achieved even modest FDR significance levels and lack of concordance between week 6 and week 12 suggests that such effects may be absent, or have substantially less impact than inter-participant or intra-participant variability. Finally, it is possible that a 12-week treatment regimen may be too short to have a meaningful impact.

In summary, we investigated the effect of atorvastatin as adjunctive immunomodulatory therapy in individuals with HIV infection on long-term ART suppression without established comorbidities or clinical indications for statin therapy, but at high risk for SNAEs. We interrogated monocyte surface markers and soluble plasma markers that are associated with and likely involved in pathogenesis of these comorbidities, as well as global monocyte gene expression profiles. This study does not provide support for atorvastatin as an adjunctive anti-inflammatory treatment in people with HIV who otherwise lack indications for statin therapy. Given the major role of SNAEs on morbidity and mortality in HIV/ART patients, further investigation of alternative agents with more promising efficacy profiles is warranted.

## SUPPLEMENTARY DATA

Supplementary materials are available at the *Pathogens and Immunity* website. Supplementary data may be provided by the authors to benefit the reader. Supplementary data are not copyedited and are the sole responsibility of the authors. Questions or comments related to supplementary materials should be addressed to the corresponding author.
