# Supplementary material for: Lack of Atorvastatin Effect on Monocyte Gene Expression and Inflammatory Markers in HIV-1-infected ART-suppressed Individuals at Risk of non-AIDS Comorbidities
Source: Pathog Immun. 2021 Aug 13;6(2):1–26. doi: 10.20411/pai.v6i2.461 (PMC8382234; doi:10.20411/pai.v6i2.461)
Supplement: Supplemental Figure 1 [file pai-6-001-s01.pdf]

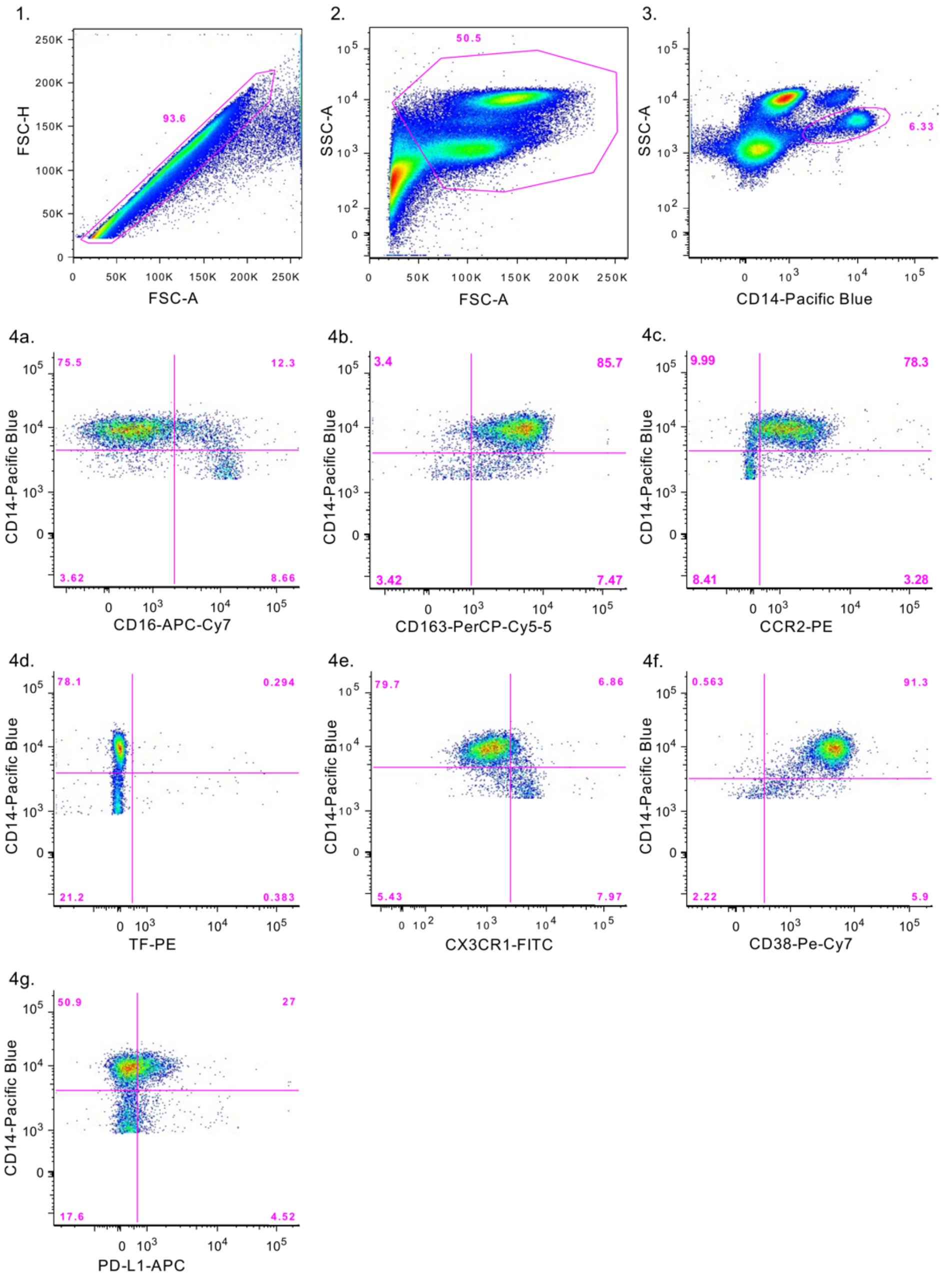

Supplementary Figure 1. Representative dot plots showing gating strategy for monocyte subset analysis: Freshly drawn whole blood stained with a cocktail of antibodies was first gated on singlets (1), followed by gating on live cells based on forward scatter (FSC) and side scatter (SSC) characteristics (2). Next, gating of total CD14+ monocytes based on SSC and CD14+ staining was done (3). Subsequently, monocyte subsets expressing CD16, CD163, CCR2, CX3CR1, CD38, TF and PD-L1 were analyzed (4a-4g). Gating for monocyte subsets was based on fluorescence minus one (FMO) staining.
