# Supplementary material for: Lack of Atorvastatin Effect on Monocyte Gene Expression and Inflammatory Markers in HIV-1-infected ART-suppressed Individuals at Risk of non-AIDS Comorbidities
Source: Pathog Immun. 2021 Aug 13;6(2):1–26. doi: 10.20411/pai.v6i2.461 (PMC8382234; doi:10.20411/pai.v6i2.461)
Supplement: Supplemental Figure 2 [file pai-6-001-s02.pdf]

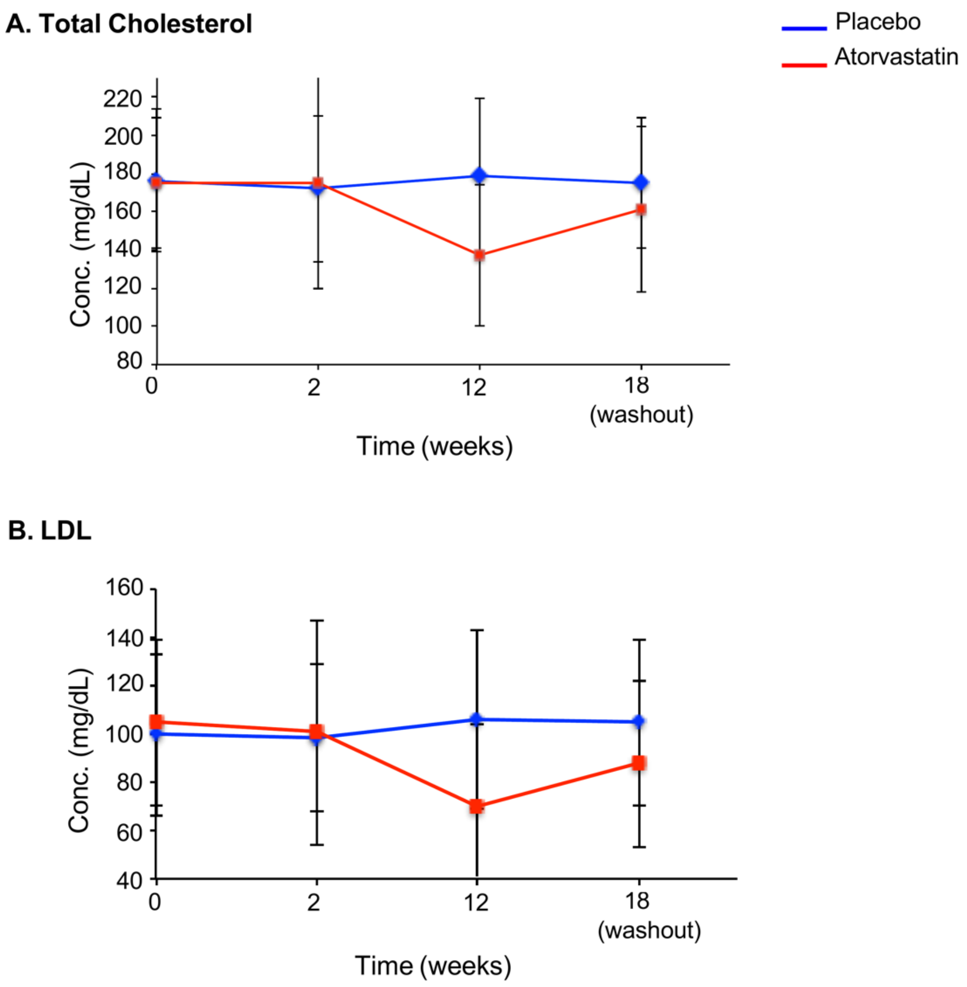

Supplementary Figure 2. Lipid changes with atorvastatin therapy: Total cholesterol (A) and low-density lipoprotein levels (LDL) (B) over 12 weeks of treatment with atorvastatin or placebo.
