# Supplementary material for: Lack of Atorvastatin Effect on Monocyte Gene Expression and Inflammatory Markers in HIV-1-infected ART-suppressed Individuals at Risk of non-AIDS Comorbidities
Source: Pathog Immun. 2021 Aug 13;6(2):1–26. doi: 10.20411/pai.v6i2.461 (PMC8382234; doi:10.20411/pai.v6i2.461)
Supplement: Supplemental Table 1 [file pai-6-001-s03.pdf]

Supplementary Table 1: Inclusion and Exclusion criteria for enrollment in study

**Inclusion criteria**
1. Chronic HIV-1 infection
2. HIV viral load ≤200 copies/mL for ≥6 months at the time of screening
3. Nadir CD4 count ≤350 (initially ≤250)
4. hsCRP >ULN
5. Karnofsky Performance score of 80 or higher
6. Age ≥18
7. Be willing to comply with study evaluations
8. Anticipated retention in geographic area for duration of study
9. On a stable antiretroviral medication regimen (no changes to treatment within 4 weeks of study entry) of NRTI/NNRTI, NRTI/RTG or NRTI/boosted PI and be willing to continue on antiretroviral therapy for the duration of the study
**Exclusion criteria**
1. Hemoglobin <10 (males) or <9 (females) g/dL ANC < 1000/µL
2. Platelet count <100,000/mm3
3. Serum creatinine > 2.0 mg/dL (177 µMol/L)
4. AST, ALT, GGT or total bilirubin > 3 times the ULN Known active liver disease
5. CPK >ULN
6. LDL chol >160
7. Known HIV-1 seroconversion within 1 year before study entry
8. Documented infection with HCV
9. Active IV drug use within 1 year prior to entry
10. Clinical indication for use of statins or contraindication to statin use
11. History of stroke, traumatic brain injury, seizures/epilepsy
12. Pregnancy or unwillingness to use contraceptive agent during study course
13. History of cancer other than successfully removed basal cell carcinomas
14. Ischemic heart disease
15. NYHA Class III or IV congestive heart failure
