# Supplementary material for: Lack of Atorvastatin Effect on Monocyte Gene Expression and Inflammatory Markers in HIV-1-infected ART-suppressed Individuals at Risk of non-AIDS Comorbidities
Source: Pathog Immun. 2021 Aug 13;6(2):1–26. doi: 10.20411/pai.v6i2.461 (PMC8382234; doi:10.20411/pai.v6i2.461)
Supplement: Supplemental Table 2 [file pai-6-001-s04.pdf]

Supplementary Table 2. Enriched pathways and functions by GSEA in HIV/ART monocytes at week 6 of atorvastatin treatment

Gene Set name	GS size	NOM P *P* value	FDR (%)	Core enrichment genes contributing to pathway enrichment
GO_PTERIDINE_CONTAINING_COMPOUND_BIOSYNTHETIC_PROCESS	17	0.0	20.6	DHFR, MTHFD2, GCH1, MTHFS, ATPIF1, GART, MTHFD1, FPGS,
GO_TETRAHYDROFOLATE_METABOLIC_PROCESS	17	0.0	42.0	DHFR, MTHFD2, GCH1, MTHFS, ATPIF1, GART, MTHFD1
ST_ERK1_ERK2_MAPK_PATHWAY	30	0.0	46.4	SOS1, SOS2, EEF2K, MAP2K2, ATF1, RPS6KA3, MAP2K1, BRAF, RPS6KA1, MOS, MKNK2, SHC1, MAPK3, CREB1, KLF6
REACTOME_SIGNALLING_TO_ERKS	31	0.0	47.4	MAPK14, SOS1, CRK, NRAS, RAF1, MAP2K2, MAP2K1, BRAF, KID!NS220, SHC1, YWHAB, MAPK3, MAPK13, KRAS, RALB, SRC, RALGDS, FRS2, PLCG1, SHC2, MAPKAPK2
REACTOME_ENOS_ACTIVATION_AND_REGULATION	19	0.006	48.8	DHFR, GCH1, HSP90AA1, NOSIP, LYPLA1, WASL, DNM2
REACTOME_SIGNALLING_TO_RAS	23	0.006	49.7	MAPK14, SOS1, NRAS, RAF1, MAP2K2, MAP2K1, SHC1, YWHAB
BIOCARTA_ERK_PATHWAY	25	0.002	49.8	SOS1, RAF1, MAP2K2, MAP2K1, RPS6KA1, MKNK2, SHC1, MAPK3, ITGB1, PDGFRA, GNAS, SRC
GO_INOSITOL_PHOSPHATE_PHOSPHATASE_ACTIVITY	19	0.003	50.6	IMPAD1, INPP5K, INPP4B, INPP5D, BPNT1, INPP5B, OCRL
GO_THYROID_GLAND_DEVELOPMENT	15	0.002	50.8	SRF, RAF1, MAP2K2, MAP2K1, BRAF, PAX8, TBX1, MAPK3, THRA
GO_REGULATION_OF_SUPEROXIDE_METABOLIC_PROCESS	16	0.002	50.8	DHFR, GCH1, CD36, AATF, FBLN5
GO_BAF_TYPE_COMPLEX	22	0.002	52.2	RB1, ARID2, SS18, ARID1A, SMARCC2, SMARCE1, PBRM1, ACTL6A, ARID1B, SMARCA4, DPF3, SMARCD1, SMARCB1
GO_UBIQUITIN_DEPENDENT_PROTEIN_CATABOLIC_PROCESS_VIA_THE_MULTIVESICULAR_BODY_SORTING_PATHWAY	17	0.003	52.3	NEDD4, VPS4B, VPS4A, RNF126, VPS25, FAM125B, VPS28, TSG101, RNF115
BONOME_OVARIAN_CANCER_POOR_SURVIVAL_UP	26	0.003	52.8	TTC37, RECK, PSD3, SEC63, PDE8A, ZBTB16, EFEMP1
GO_SENSORY_PERCEPTION_OF_TASTE	22	0.002	54.1	CA6, PKD2L1, TAS2R43, CD36, TAS2R39, CST4, GNAT1, TAS2R10, TAS2R3, ITPR3, PLCB2
BIOCARTA_IL6_PATHWAY	21	0.001	55.7	SRF, IL6R, SOS1, RAF1, JAK2, MAP2K1, CSNK2A1, SHC1, MAPK3
CAFFAREL_RESPONSE_TO_THC_UP	30	0.0	56.2	IPO7, SEC31A, RAB5A, CPEB4, SLC3A2, SERINC1, MCCC1, ZNF451, RPS2, NUCB2, YY1AP1, ZRANB2, USP3
BIOCARTA_EIF_PATHWAY	16	0.001	58.1	EIF4G2, EIF6, EEF2K, EIF2S3, EIF1AX, EIF4G1, EIF4G3
KEGG_TASTE_TRANSDUCTION	22	0.0	60.8	PRKX, TAS2R43, TAS2R39, PRKACG, CACNA1B, PRKACB, TAS2R10, TAS2R3, ITPR3, PLCB2, GRM4, ADCY6, GNAS
REACTOME_ARMS_MEDIATED_ACTIVATION	15	0.0	64.6	CRK, NRAS, RAF1, MAP2K2, MAP2K1, BRAF, KIDINS220, YWHAB, MAPK3, KRAS
GO_LIGAND_DEPENDENT_NUCLEAR_RECEPTOR_BINDING	20	0.0	73.7	ARID1A, SMARCE1, NCOA2, JUND, UBA3, NCOA6, NCOA1

GS size: Gene set size (number of genes); NOM p-value: nominal *P* value; FDR: false discovery rate. The top 20 enriched gene sets are listed in order of increasing FDR. Only one gene set was enriched at FDR<25%. A total of 399 gene sets are significantly enriched at nominal *P* value <1% and 1434 at nominal *P* value <5%.
