# Supplementary material for: Lack of Atorvastatin Effect on Monocyte Gene Expression and Inflammatory Markers in HIV-1-infected ART-suppressed Individuals at Risk of non-AIDS Comorbidities
Source: Pathog Immun. 2021 Aug 13;6(2):1–26. doi: 10.20411/pai.v6i2.461 (PMC8382234; doi:10.20411/pai.v6i2.461)
Supplement: Supplemental Table 3 [file pai-6-001-s05.pdf]

Supplementary Table 3. Enriched pathways and functions by GSEA in HIV/ART monocytes at week 12 of atorvastatin treatment

Gene Set name	GS size	NOM *P* value	FDR (%)	Core enrichment genes contributing to pathway enrichment
GO_PROTEIN_LIPID_COMPLEX	23	0.003	41.6	LPA, ABCA1, LDLR, SORL1, PCYOX1, wwLSR, LCAT, SAA1, HPR
BIOCARTA_TNFR1_PATHWAY	29	0.001	42.0	PAK1, MAP3K7, PRKDC, LMNB1, DFFB, RB1, LMNA, PAK2, TRADD, CASP8, CASP2, TNF, CRADD, MADD
LIU_BREAST_CANCER	21	0.003	43.2	FAU, GGTLC1, CTSB, DHX16, ITGB1, NCOR1, LOC100129827
WALLACE_PROSTATE_CANCER_UP	18	0.003	43.2	AMACR, BDH1, TPD52, SOX4, PPP3CA, FASN
GO_TETRAPYRROLE_BIOSYNTHETIC_PROCESS	26	0.001	43.7	UROD, FXN, PPOX, ABCB6, SLC11A2, ATPIF1, COX10, TMEM14C, TSPO, SUCLA2
GO_NEGATIVE_REGULATION_OF_RESPONSE_TO_OXIDATIVE_STRESS	29	0.002	44.3	GPR37, VNN1, MET, AKT1, TRAP1, HSPH1, SOD2, ATF4, MAPK7, HIF1A, PSAP, TXNDC3, NFE2L2
GO_PROTEIN_KINASE_B_SIGNALING	25	0.001	45.0	MERTK, AKT1, EP300, CD40, GAS6, AXL, SESN2, PIK3CA, TNF, PLK3, AKT2, IGF1
HORTON_SREBF_TARGETS	24	0.001	45.6	RDH11, LDLR, FDPS, TM7SF2, AACS, ACACA, ACSS2, ALDOC, LSS, FASN, ELOVL6
GO_MYOTUBE_CELL_DEVELOPMENT	20	0.004	46.2	BIN3, SEPN1, PPP3CA, CAV2, RYR1, IGF1, MYOG, SMYD3, DNER, LOC375190, STAC3, P2RX2
GO_GENETIC_IMPRINTING	19	0.005	46.3	ARID4A, MECP2, DIRAS3, ARID4B, CTCF, KCNQ1
GO_POSITIVE_REGULATION_OF_RESPONSE_TO_BIOTIC_STIMULUS	31	0.0	46.5	LY96, PUM2, ANKRD17, NOD1, MAPK3, DHX58, ZC3HAV1, PUM1, HSPD1, DDX60, LRSAM1
GO_NEGATIVE_REGULATION_OF_OXIDATIVE_STRESS_INDUCED_INTRINSIC_APOPTOTIC_SIGNALING_PATHWAY	18	0.001	47.1	VNN1, AKT1, TRAP1, HSPH1, SOD2, MAPK7, HIF1A, TXNDC3, NFE2L2
BIOCARTA_HCMV_PATHWAY	17	0.005	47.6	MAP2K2, AKT1, RELA, PIK3CG, MAPK3, RBI, MAPK14, MAP2K3, PIK3CA
GO_VASOCONSTRICTION	16	0.0	50.1	P2RX1, SLC8A1, PIK3C2A, TRPM4,
SIG_REGULATION_OF_THE_ACTIN_CYTOSKELETON_BY_RHO_GTPASES	25	0.0	57.1	PAK1, AKT1, RPS4X, GDI2, ACTG1, ROCK1, PFN2, PAK2, ACTR2, VASP, CDC42, FLNA
ZHANG_RESPONSE_TO_CANTHARIDIN_UP	16	0.003	58.0	ZFX, TRAF3, NAMPT, TRIM13, BCL10, NFE2L2, TGFBR2, NFIC, FPR1
SHARMA_PILOCYTIC_ASTROCYTOMA_LOCATION_UP	17	0.0	64.1	LY96, TRUB1, GPC6, SBNO1, TTC9, APOBEC3G, C14orf39, CASP7
MATZUK_SPERMATOGONIA	15	0.0	69.3	P2RX1, LIMK2, KIT, CYP19A1, NANOS2, ZBTB16, ETV5, SYCP2, APAF1, BMP8B
GO_REGULATION_OF_GENE_EXPRESSION_BY_GENETIC_IMPRINTING	15	0.0	90.9	ARID4A, MECP2, DIRAS3, ARID4B, CTCF, KCNQ1
COLLER_MYC_TARGETS_UP	23	0.0	100	POLR2H, TRAP1, ASS1, HSPD1, NAMPT, IARS, CEBPZ

GS size: Gene set size (number of genes); NOM *P* value: nominal *P* value; FDR: false discovery rate. The top 20 enriched gene sets are listed in order of increasing FDR. There were no gene sets enriched at FDR<25%. A total of 443 gene sets are significantly enriched at nominal p value <1% and 1461 at nominal *P* value <5%.
